# Foods from the wild: Local knowledge, use pattern and distribution in Western Nepal

**DOI:** 10.1371/journal.pone.0258905

**Published:** 2021-10-21

**Authors:** Dhruba Khakurel, Yadav Uprety, Łukasz Łuczaj, Sangeeta Rajbhandary

**Affiliations:** 1 Chungnam National University, Daejeon, South Korea; 2 Central Department of Botany, Tribhuvan University, Kirtipur, Kathmandu, Nepal; 3 Institute of Biology and Biotechnology, University of Rzeszów, Rzeszów, Poland; University of Sao Paulo, BRAZIL

## Abstract

Locally harvested wild edible plants (WEPs) provide food as well as cash income for indigenous peoples and local communities, and they are of great importance in ensuring local food security. However, their uses and availability are poorly documented. This study aimed to enumerate WEP diversity and status of WEPs in a part of the Annapurna Conservation Area, Sikles region, where the population is dominated by the *Gurung* community. Ethnobotanical data were collected using guided field walks, semi-structured interviews, and field observation. The informant consensus method was employed and group discussions were conducted for triangulation of the information. Free listing and identification tests were performed to assess the knowledge of the informants. Both descriptive statistics and quantitative ethnobotanical methods were used for data analysis. A total of 72 wild food species belonging to 46 families and 61 genera were reported from the study area. Asparagaceae and Rosaceae were the dominant families, and herbs were the dominant life form. Fruits (34 species) were the most frequently used plant parts, followed by young shoots (16 species). Most edible plants were consumed in summer and during rainy seasons. While the age and type of informants had an influence on the number of enumerated plants, gender did not. Key informants and people aged 30–45 reported more species than other groups of respondents. Most of the knowledge about the use of WEPs was acquired from parents and relatives. The consumption of these plants was attributed to diversifying cuisine, spicing staple food, nutri-medicinal values, and cultural practices. People perceived the availability of WEPs to be gradually decreasing. However, WEPs are still abundant and diverse in the study area, and knowledge on their use is well-preserved. These resources provide food and nutrients to local people and can also be a source of cash income. Therefore, the documented information on WEPs may serve as baseline data for further studies on nutritional values and provide guidelines for safe collection. The results also revealed that many wild species are under growing pressure from various anthropogenic factors, suggesting effective community engagement is required for their conservation.

## Background

Plant biodiversity provides human beings with all kinds of ecosystem goods and services. Among them, provisioning services such as food, fodder, medicine, timber and fuelwood are the most fundamental for survival [1,2]. In most parts of the developing world, humans rely heavily on local environmental resources, especially wild plants, for daily subsistence and health care. Traditional knowledge on the use of these resources is regarded as a means for adaptation during periods of hardship [3]. Therefore, studies of human interactions with plants are relevant to many global issues, including food security, climate change, conservation biology, and human health [1,3]. Local communities have developed preservation methods including fermenting, pickling, salting and drying edible wild plants to be used throughout all seasons [4–6]. Many traditional societies have depended on wild-growing plants in their diets for thousands of years, and many people continue to rely on these species to meet at least part of their daily nutritional needs. Wild harvested plant foods include: roots and other underground parts as tubers; young shoots and leafy greens parts; fruits, berries and other fleshy fruits; dry fruits and seeds; tree saps and resins; flowers; edible fungi; algae, and other species. The use of any of these species requires special cultural knowledge regarding harvesting, preparation, cooking, and other forms of processing [7–10].

The term “wild edible plants” (WEPs) refers to species harvested from wild plants or to plants growing spontaneously in an area, i.e. without being cultivated, including native species as well as introduced species that have been naturalized and are ingested as food [11–13]. The collection and consumption of WEPs has been “a way of life for many rural populations throughout the world, supplementing their dietary requirements [14,15]. Knowledge on WEPs is of high direct-use value helping both to reduce the necessity of buying marketed alternatives and achieve food security [16,17].

Although inexpensive, WEPs are a rich source of antioxidants, vitamins, fiber, and minerals, and often serve as dietary supplements or as famine food in times of scarcity. Some species are also a good source of calories. Moreover, some edible plants–food medicines—are deliberately consumed for medicinal purposes [18]. WFPs have considerable potential for the development of new crops through domestication and provide a genetic reserve for hybridization and selection [19].

Many uncultivated plant species are used by rural households in Nepal. The few studies on this subject either focused on a particular ethnic group [20,21] or provided an overview of WEP use across large regions [16,22–30]. However, comprehensive studies about the availability, status, and contribution of WEPs to livelihoods are scarce. Particularly in mountainous areas of high biodiversity, species that are interesting in terms of endemism and multiple uses can be documented. Sometimes such studies also find ancient detoxification procedures [31]. The Himalayas are definitely a place where traces of ancient wisdom on the use of WEP can be found [32]. Traditional knowledge on the use of medicinal plants from the Sikles area of Kaski district has been previously reported [33,34], but no documentation of wild food species has been made. This study fills the gap by documenting the wild food species, culture and livelihood of the indigenous people of Sikles and adjoining villages. The present study seeks to answer the following questions: i) How are WEPs distributed in the study area and across seasons, which plant part(s) is (are) used, and do they also have medicinal uses? ii) How is knowledge of WEPs distributed between generations and genders? iii) What conservation measures are practiced or could be practiced for the most useful species?

The study was carried out in culturally diverse areas of equal importance from an ecological and biodiversity point of view. The area lacked comprehensive inventories of WEPs that could in turn support planning for biodiversity conservation and sustainable use. There are many reasons to study WEPs, not only to preserve them from being forgotten but also to conserve their precious genetic resources for the wellbeing of our future generations. Therefore, detailed study on WEPs is needed to understand their contribution to local diets, construct priority species lists, and evaluate possibilities for domestication or propose sustainable harvesting techniques.

## Materials and methods

### Study area

**Fig 1:**
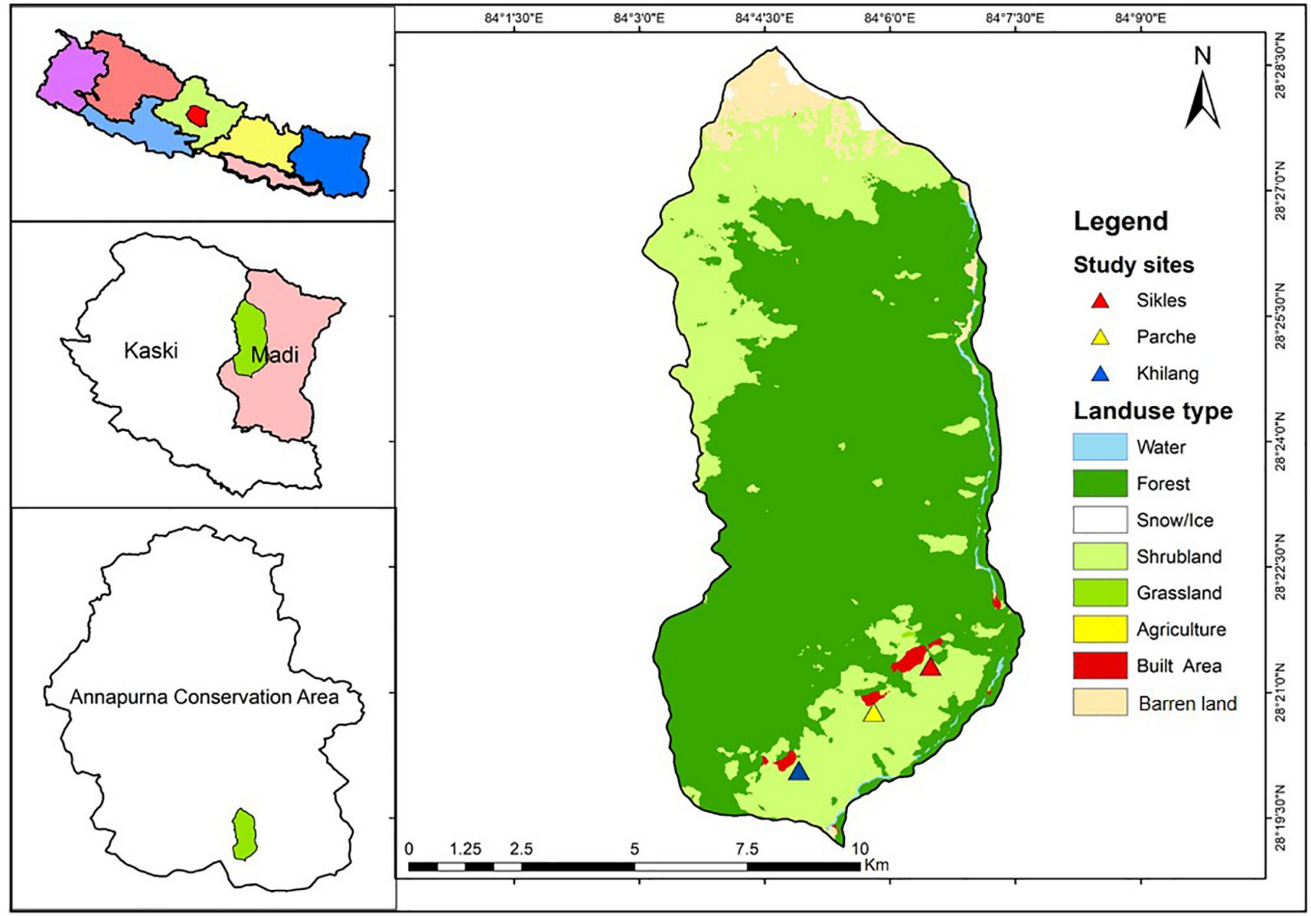
Location map of the study area. The upper left represents a map of Nepal with the study district highlighted in red; the middle left is an outline of the district, with Madi Rural Municipality in light pink and the study area shadowed in green; the lower left represents Annapurna Conservation Area and the image on the right shows the exact location of the villages together with the land use pattern.

This study was carried out with the communities of Khilang, Parche and Sikles villages of Madi Rural Municipality (Ward no. 1) in Kaski district, Gandaki Province of western Nepal. Sikles is the largest village of the three, situated on a mountainside (Fig 1) Geographically, the study area is located around 28°28’N- 28°47’N / 84°00’E -84°42’E at an altitude ranging from 1,400 to 4,000 m. The area is a part of Annapurna Conservation Area (ACA), the largest conservation area in Nepal.

Out of the total 2,500 inhabitants of these villages, about 70% are the members of the *Gurung* community [35]. The *Gurungs* are one of the major ethnic groups of Nepal. Having traditionally lived in mid- to high-hills, they possess many generations of experience with the local vegetation [36,37]. This community has unique adaptation to different environmental conditions, as revealed by their culture and livelihoods [38].

The actual population residing in the villages is lower than census data because of absentee population (reported at around 700 [35]) resulting from migration to big cities and foreign countries. Agriculture is the main livelihood strategy; some people also engaged in small businesses as the study area is one of the main tourist destinations in the region. A primary health care center provides basic health care facilities and complicated cases are usually referred to the hospitals in Pokhara, a major city of Kaski situated an approximately five-hour drive away. Access to the city is difficult during the rainy season, as the roads are gravel or earth constructions. Easy access to the city would be boon to local livelihood, improving access to tourists and enabling the surplus of wild edibles to be sold in the city.

Altitudinal and climatic variations are responsible for the high diversity of plants in the study area, which consist of upper subtropical vegetation to lower alpine vegetation with *Alnus* forests, mixed forests, broad leaved forests, evergreen forests, shrubland and grasslands [39]. These forests cover some 54% of the land. Since the study area lies near the Kali Gandaki River that separates the Eastern and Western Himalayan floristic regions, typical assemblages of both floral regions can be found in the study area [40]. The climate is influenced by monsoons but varies seasonally and remains rather cool throughout the year with heavy snowfall once or twice a year in elevations above 2,000 m. The seasons are broadly divided into winter (December-February), spring (March-May), summer (June- August) and autumn (September-November).

### Methods

#### Prior informant consent

A research permit was given by the Department of National Parks and Wildlife Conservation and the Annapurna Conservation Area Project after discussing the objectives of the study with the authorities. In the villages where research was to be undertaken, prior oral informed consent for recording and disseminating local knowledge was obtained by explaining the aim of the study to the community leaders, including the Ward President. Respondents were ensured that their traditional knowledge would be used only for research purposes. As the area was one of the pilot sites for the access and benefit sharing (ABS) project of the Government of Nepal and IUCN, the communities were further ensured that the ABS process and laws would apply in the case of further research and development [41].

#### Sampling design and informant selection

A reconnaissance survey was made from February 15 to 28, 2018, and three study sites based on altitude and locality were purposively selected. Data collection was performed between 25 June and 5 August, 2018. A total of 62 informants (43% female and 57% male) between the age of 17 and 75, at least fifteen individuals from each village were interviewed. Additionally, eight key informants were interviewed using purposive sampling technique, making sure that at least two key informants from each study site were included. The key informants included specialists aged 25 to 75 years and were selected with the help of local people and community leaders. Fifty-seven percent of the respondents had no formal education, 21% had primary level education, 10% had secondary level education and 12% had higher education. The respondents were comprised of 90% farmers, including 12% living mostly in high-land pastures for rearing livestock, whereas 10% were part of small business mainly involved in the collection and sale of medicinal plants.

Semi-structured interviews, guided field walks, focus group discussions, and field observation were the approaches applied to gather the data [42–44]. Questionnaires were administrated in the Nepali language (Supporting information file). First, a brief group discussion was held with informants at each sample site, and free listing of the WEPs was performed. An identification test of specimens was performed with the help of photographs and plant specimens collected together with the informants. Other required information was collected from individual interviews as stipulated in the questionnaires. Three age groups were distinguished: 15–30 years, 30–45 years and above 45 years old.

#### Plant specimen collection and identification

Along with herbarium collection during guided field walks, other field activities included taking notes about the plants and the associated traditional knowledge with preliminary identification of the family and sometimes up to the species level. Each specimen (except for a few common species that were not collected) was given a collection number and a scientific and/or local name where possible. Information was also captured with photographs to document the sites, individual plants and edible parts. The specimens were identified with the help of standard literature [45–47]. A comparison of the specimens was also made with specimens deposited at the National Herbarium (KATH) and Tribhuvan University Central Herbarium (TUCH) to ensure taxonomic determination. Nomenclature follows the catalogue of life (https://www.catalogueoflife.org/col/search/). The voucher specimens were deposited at TUCH.

### Data analysis

A Microsoft Excel spreadsheet was employed for organizing the ethnobotanical data. The collected information on WEPs was quantitatively analyzed using the index of relative frequency of citation (RFC):

RFC=FC/NWhere,0<RFC<1

This index indicates the local importance of each species, assessed by the frequency of citation (FC, the number of informants mentioning the use of the species) divided by the total number of informants participating in the survey (N), without considering use-categories [48–50]. It may vary from 0 to 1; consequently, a RFC value close to 1 means that a species is very important from a cultural and traditional point of view. Following this method, each informant (n = 8, all key informants + 2 other informants) was asked to think of, order, and rank each plant based on their personal preference and the usefulness of the species. Preference ranking and RFC were performed to analyze the most popular and preferred species, at least in the context of the people who used them to diversify cuisine in the area [44,50,51].

Mann Whitney U test was performed to test the significance of difference between genders (male and female). Regression analysis was also performed to find the relationship between enumerations of species and the age of the respondents.

## Results

### Diversity of wild edible plants and fungi

**Fig 2:**
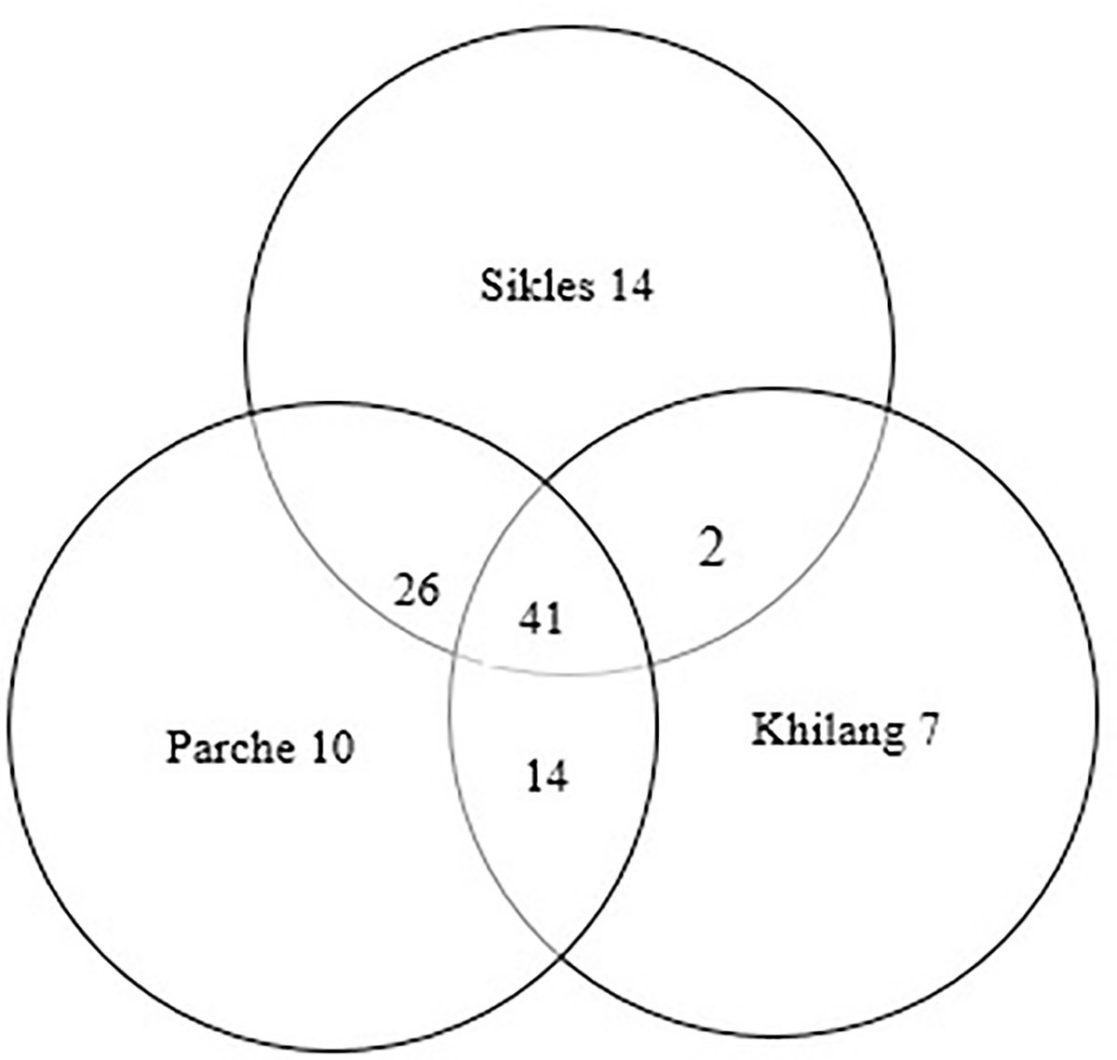
A Venn diagram showing the number of WEPs reported from informants of three villages. The species included 65 angiosperms and six pteridophytes with six species each belonging to Asparagaceae and Rosaceae, four each to Polygonaceae and Urticaceae, and three each to Berberidaceae and Begoniaceae (Fig 3).

This study reported 72 wild food species belonging to 46 families and 61 genera (Table 1). Forty-one species were common species reported by informants from all three villages, whereas 14 species were reported from Sikles only, 10 species from Parche only, and 7 species from Khilang only (Fig 2).

**Fig 3:**
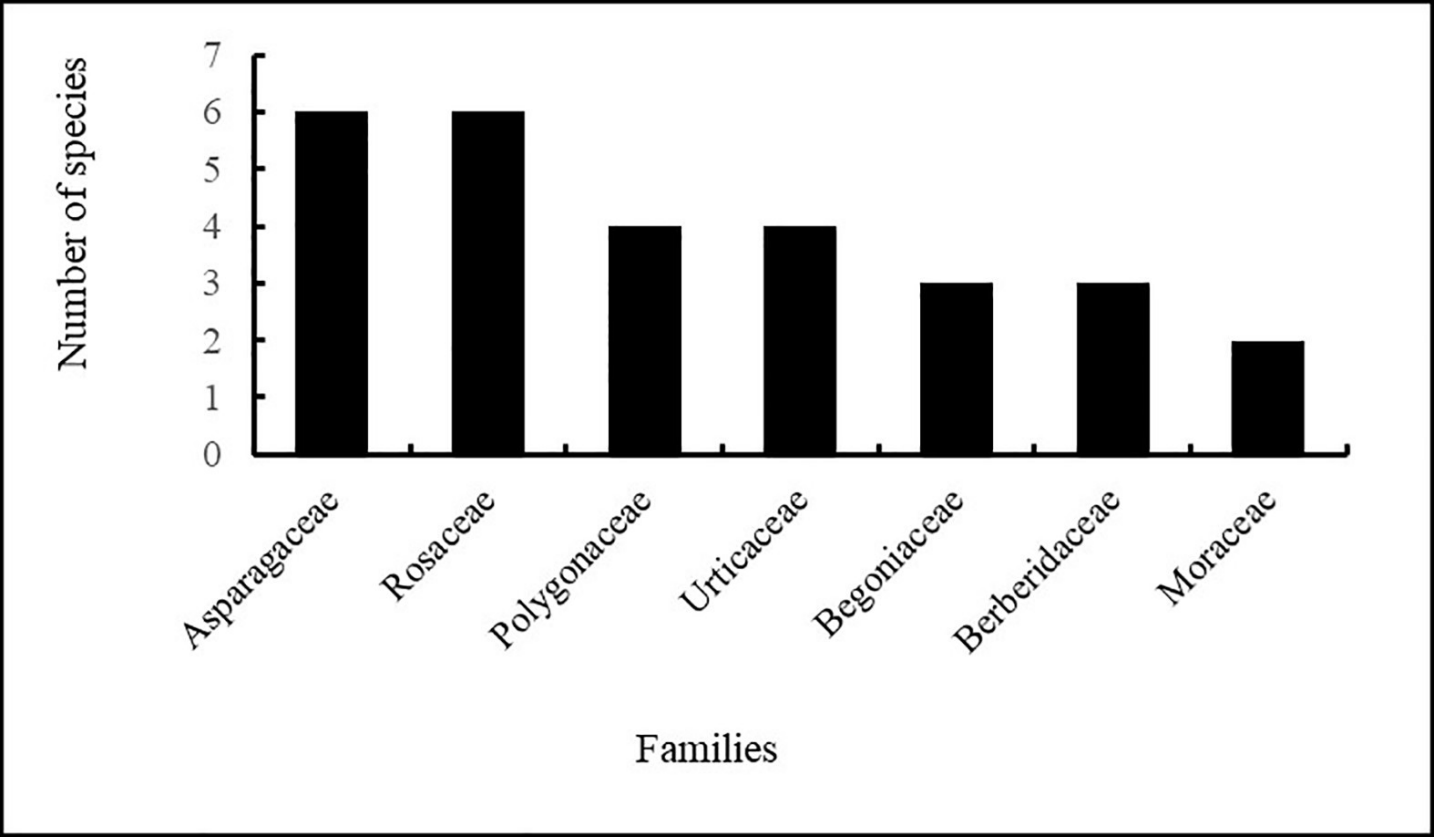
Dominant families of wild edible plants.

**Table 1:** Names, life forms, harvesting time, parts used, local uses and additional local uses of wild edible plants.

S.N.	Scientific name	Collection no.	Common name	Gurung name	Family	Life form*	Harvesting time	Parts used^¥^	Use (edible only)	Additional use	RFC^ɸ^ (n = 70)
1	*Allium wallichii* Kunth	KSD97	Ban lasun	Ban nhoo	Amryllidaceae	H	July-October	L	Dried leaves used as a condiment in curries and pickles.	Vegetable is considered a tonic and used to treat coughs and colds.	0.79
2	*Alsophila spinulosa* Wall. ex Hook.	KSD73	Chatte niguro	Motana	Cyatheaceae	T	June-July	Ys	Young shoots used to make pickles.	Used as an ornamental.	0.54
3	*Arisaema costatum* (Wall.) Mart.	KSD37	Sarpa makai	Tobyo	Araceae	H	April-July	L	Young leaves used as vegetables.	-	0.19
4	*Asparagus racemosus* Willd.	KSD20	Kurilo	Puchu touru	Asparagaceae	S	June-July	Ys	Young shoots used as vegetables.	Vegetable is considered a tonic and high- nutritional food.	0.97
5	*Begonia dioica* Buch.- Ham. ex D.Don	KSD91	Magar kanche		Begoniaceae	H	July-August	Pt, L	Stem and leaves eaten raw or used to made pickles.	-	0.63
6	*Begonia palmata* D.Don	KSD99	Magar kanche	Khagayo	Begoniaceae	H	June-July	Pt	Eaten raw.	Stem used to treat stomach pain.	0.36
7	*Begonia picta* Sm.	KSD98	Magar kanche	Prugyu	Begoniaceae	H	July-August	Pt	Eaten raw and also use to make pickles.	Used for constipation.	0.77
8	*Berberis aristata* DC.	KSD06	Chutro	Tishya	Berberidaceae	S	May-June	F	Fruits	Bark used to treat eye problems.	0.89
9	*Berberis concinna* Hook.f.	KSD791	Lekh chutro	Lekh tishya	Berberidaceae	S	July-August	F	Fruits	-	0.19
10	*Berberis napaulensis* (DC.) Spreng.	KSD63	Jamane mandro	Komee	Berberidaceae	S	Feburary- April	F	Fruits	-	0.23
11	*Brucea javanica* (L.) Merr.	KSD305	Bhakiamilo	Tiuru	Simaroubaceae	T	November- March	F	Eaten raw and also dried fruit powder used in pickles.	Fruit used to control diarrhea.	0.53
12	*Cannabis sativa* L.	KSD47	Ganja	Vamm	Cannabaceae	H	July-September	Se	Used to make pickles.	Leaf and shoot powder used to treat coughs and diarrhea.	0.20
13	*Castanopsis indica* (Roxb. ex Lindl.) A.DC.	KSD53	Katus	Kasi	Fagaceae	T	September-November	F	Fruits	Bark paste used as dye.	0.60
14	*Chenopodium album* L.	KSD399	Bethe	Lainu	Amaranthaceae	H	July-August	Ys, Se	Young shoots used as vegetables and seeds used to make pickles.	Vegetables eaten to treat stomach problems.	0.36
15	*Chlorophytum nepalense* (Lindl.) Baker	KSD114	Danti sag		Asparagaceae	H	June-September	Tu	Fresh tuber eaten raw.	-	0.17
16	*Choerospondias axillaris* (Roxb.) B.L.Burtt & A.W. Hill	KSD82	Lapsi	Kalah	Anacardiaceae	T	September-January	F	Fruits	-	0.36
17	*Cinnamomum tamala* (Buch.-Ham.) Th. G. G. Nees	KSD55	Tejpat	Lepu	Lauraceae	T	October-December	L, Ba	Used as spice.	Spice	0.84
18	*Cirsium verutum* (D. Don) Spreng.	KSD69	Thakailo	Popuche	Asteraceae	H	June-August	Rt, Ys	Eaten raw.	Root used to treat fever.	0.40
19	*Coriaria nepalensis* Wall.	KSD38	Machino		Coriariaceae	S	March- May	F	Fruits	-	0.13
20	*Dactylorhiza hatagirea* (D. Don) Soó	KSD58	Panchaule	Yori-nghee	Orchidaceae	H	July-September	T	Eaten boiled.	Tubers are used as a tonic, externally to treat burns, cuts and wounds and are eaten to cure stomach problems.	0.09
21	*Debregeasia longifolia* (Burm. f.) Wedd.	KSD302	Tushaare		Urticaceae	T	October-November	F	Fruits	-	0.54
22	*Dendrocalamus hamiltonii* Nees & Arn. ex Munro	KSD72	Bans	Ri dhu	Poaceae	S	September-October	Ys	Young shoots used as vegetables.	Fiber yielding.	0.33
23	*Dioscorea bulbifera* L.	KSD22	Ban tarul	Ban temee	Dioscoreaceae	C	November-December	Tu	Tubers used as vegetables.	Religious use.	0.54
24	*Dioscorea deltoidea* Wall. ex Griseb.	KSD403	Vaykur	Temee	Dioscoreaceae	C	November-February	Tu	Tubers used as vegetables.	-	0.66
25	*Diplazium esculentum* (Retz.) Sw.	KSD610	Pani niguro	Lauta	Athyriaceae	S	April-June	Ys	Young shoots used as vegetables.	-	0.76
26	*Elaeagnus infundibularis* Momiy.	KSD11	Guheli	Turu	Elaeganaceae	T	June-August	F	Fruits	-	0.20
27	*Ficus auriculata* Lour.	KSD187	Nimaro	Toubu dhu	Moraceae	T	May-August	F	Fruits	-	0.41
28	*Ficus semicordata* Buch. ex J.E. Smith	KSD186	Khanayo	Mowa dhu	Moraceae	T	June-July	F	Fruits	Religious use.	0.40
29	*Fragaria nubicola* Lindl.	KSD344	Bhui ainselu	Sa palaha	Rosaceae	H	May-August	F	Fruits	-	0.49
30	*Girardinia diversifolia* (Link) Friis	KSD01	Allo	Puwa/ Ni polu	Urticaceae	S	June-July	Ys	Young shoots used as vegetables.	Fiber yielding	0.20
31	*Hemipragma heterophylla* Wall.	KSD106	Bhui kafal		Plantaginaceae	H	June-August	F	Fruits	-	0.21
32	*Himalayacalamus brevinodus *Stapleton	KSD42	Malinge nigalo	Plomu mo	Poaceae	S	June-August	Ys	Young shoots used as vegetables.	Fiber yielding	0.81
33	*Houttuynia cordata* Thunb.	KSD122	Gandhe	Fitta dhu	Saururaceae	H	July-August	Rt	Root used to make pickles.	-	0.70
34	*Juglans regia* L.	KSD43	Okhar	Kathu	Juglandaceae	T	September-October	F	Fruits	Bark used to treat skin problems.	0.37
35	*Koenigia polystachya* (Wall. ex Meisn.) T.M.Schust. & Reveal	KSD710	Thotne		Polygonaceae	S	July-August	Ys	Young shoots used as vegetables.	-	0.46
36	*Lindera neesiana* (Wall. ex Nees) Kruz	KSD33	Siltimur	Kudu	Lauraceae	T	August-September	F	Fruits	Fruit used to treat stomach and gastric problems. Spice. Used in tea.	0.94
37	*Maianthemum purpureum* (Wall.) LaFrankie	KSD85	Sikaaree Saag	Narpunta	Asparagaceae	H	June-July	Ys	Young shoots used as vegetables.	-	0.34
38	*Morchella esculenta* (L.) Pers.	KSD89	Guchi chyau		Morchellaceae	H	June-July	Sh	Fruiting body edible.	Considered as high nutritional food.	0.31
39	*Myrica esculenta* Buch.-Ham. ex D.Don	KSD714	Kafal		Myricaceae	T	November-May	F	Fruits	Bark paste eaten to treat stomachache.	0.37
40	*Nephrolepis cordifolia* (L.) Presl	KSD625	Pani amala		Nephrolepidaceae	H	June-July	T	Tuber eaten raw.	Used as ornamentals.	0.59
41	*Ophioglossum reticulatum* L.	KSD107	Jibre sag	Pareta	Ophioglossaceae	H	June-July	St, L	Stem and leaves used as vegetables.	-	0.34
42	*Ophiopogon clarkei* Hook.f.	KSD109	Kali gedi		Asparagaceae	H	June-July	F	Fruits	-	0.31
43	*Osbeckia stellata* Buch.-Ham. ex D. Don	KSD442	Angeri		Melastomataceae	S	October-December	F	Fruits	-	0.37
44	*Paris polyphylla* Sm.	KSD57	Satuwa	Satuwa	Melanthiaceae	H	July-August	Fl, F	Flowers and fruits eaten.	Rhizome used to treat burns, cut and wounds.	0.20
45	*Phytolacca acinosa* Roxb.	KSD60	Jaringo sag	Olita	Phytolaccaceae	S	June-July	Ys	Young shoots used as vegetables.		0.56
46	*Piper mullesua* Buch.- Ham. ex D. Don	KSD42	Chiya ghass	Sindri	Piperaceae	H	July- August	F	Fruits	Fruits used in fermentation.	0.40
47	*Polygonatum cirrhifolium* (Wall.) Royle	KSD21	Khirimla	Khirimla	Asparagaceae	H	May-June	Ys	Young shoots used as vegetables.	Rhizome considered a tonic.	0.66
48	*Polygonatum punctatum* Royle ex Kunth	KSD93	Thulo khirimla	Khirimla	Asparagaceae	H	May-June	Ys	Young shoot used as vegetables.	-	0.37
49	*Pouzolzia sanguinea* (Bl.) Merr.	KSD75	Chiple	Pleta chi	Urticaceae	H	July-September	Rt	Root powder used to make bread (*Sel roti*).	Root paste used as soap.	0.73
50	*Prunus cerasoides* D. Don	KSD16	Paiyun	Chyarbu	Rosaceae	T	December-February	F	Fruits	Religious use.	0.37
51	*Pteridium revolutum* (Bl.) Nakai	KSD612	Badhaure niguro	Lakhuto	Dennstaedtiaceae	S	June-July	Ys	Cooked young shoots eaten as vegetables.	-	0.30
52	*Pyracantha crenulata* (Roxb. ex D.Don) M.Roemer	KSD26	Ghangaru	Chaido	Rosaceae	S	August-November	F	Fruits	-	0.51
53	*Pyrularia edulis* (Wall.) A. DC.	KSD317	Amphi	Yomi	Santalaceae	T	July-November	F	Fruits	Oil obtained from seed is used to control cracked skin.	0.54
54	*Rheum acuminatum* Hook. fil. & Thoms.	KSD771	Padamchal		Polygonaceae	H	June-July	St, L	Stem and leaf used to make pickles.	-	0.40
55	*Rheum australe* D.Don	KSD772	Padamchal	Pudumchalne	Polygonaceae	H	June-July	St, L	Stem and leaf eaten raw and used to make pickles.	Rhizome paste used to treat fractures.	0.41
56	*Rhododendron arboreum* Sm.	KSD100	Lali gurans	Pori	Ericaceae	T	May-June	Fl	Flowers		0.69
57	*Rubus ellipticus* Sm.	KSD87	Aaiselu	Palaha	Rosaceae	S	May-July	F	Fruits	Root paste eaten to cure fever.	0.91
58	*Rubus nepalensis* (Hook.f.) Kuntze	KSD86	Bhui aaiselu	Sa palaha	Rosaceae	H	May-June	F	Fruits	-	0.34
59	*Rubus paniculatus* Sm.	KSD92	Kalo aaiselu	Mlo palaha	Rosaceae	S	May-July	F	Fruits	-	0.37
60	*Rumex nepalensis* Spreng.	KSD62	Halhale	Olmi	Polygonaceae	H	April-May	L	Leaves used as vegetables.	-	0.86
61	*Saurauia napaulensis* DC.	KSD19	Gogan	Pleshi dhu	Actinidiaceae	T	April-August	F	Fruits	-	0.30
62	*Scurrula parasitica* L.	KSD52	Lisso	Mephu dhu	Loranthaceae	S	October-January	F	Fruits	-	0.11
63	*Smilax aspera* L.	KSD27	Kukurdaino	Neeri	Smilacaceae	C	June-July	Ys	Young shoots used as vegetables.	Religious use.	0.23
64	*Solanum nigrum* L.	KSD304	Kali gedi	Taujamai	Solanaceae	H	July-November	F	Fruits	-	0.24
65	*Solena amplexicaulis* (Lam.) Gandhi	KSD307	Gol kakri	Toju	Cucurbitaceae	C	August-September	F	Fruits	-	0.51
66	*Stauntonia angustifolia* (Wall.) Christenh.	KSD04	Gofla	Malkaji	Lardizabalaceae	C	July-August	F	Fruits	-	0.63
67	*Tectaria coadunata* (Wall. ex Hook. & Grev.) C. Christensen	KSD107	Kalo neuro		Tectariaceae	H	May-June	Ys	Young shoots used as vegetables.	-	0.80
68	*Trichosanthes tricuspidata* Lour.	KSD776	Indrenee		Cucurbitaceae	C	August-October	Se	Eaten after roasted.	Seeds eaten to control vomiting.	0.37
69	*Urtica dioica* L.	KSD02	Sisno	Polu	Urticaceae	H	June-July	Ys	Young shoots used as vegetables.	Root juice used in fever. Fiber yielding	0.63
70	*Vaccinium nummularia* Hook. Fil & Thoms. ex C.B. Cl.	KSD101	Kali gedi		Ericaceae	H	July-August	F	Fruits	-	0.06
71	*Viburnum mullaha* Buch.-Ham. ex D. Don	KSD105	Molo	Hera	Adoxaceae	T	October-November	F	Eaten raw. Dried fruit powder used in pickles.	-	0.84
72	*Zanthoxylum armatum* DC.	KSD311	Timur	Prumo	Rutaceae	T	August-October	F	Fruits used as spice.	Fruit powder eaten to treat stomach pain. Religious use.	0.93

*Life From: H: Herb; C: Climber; S: Shrub; T: Tree.

^¥^Parts Used: Ba-Bark; L- Leaf; Fl-Flower; F-Fruit; St-Stem; Pt-Petiole; Tu-Tuber; Rt-Root; Se-Seed; Ys-Young shoot.

^ɸ^RFC = Relative frequency of citation.

**Fig 4:**
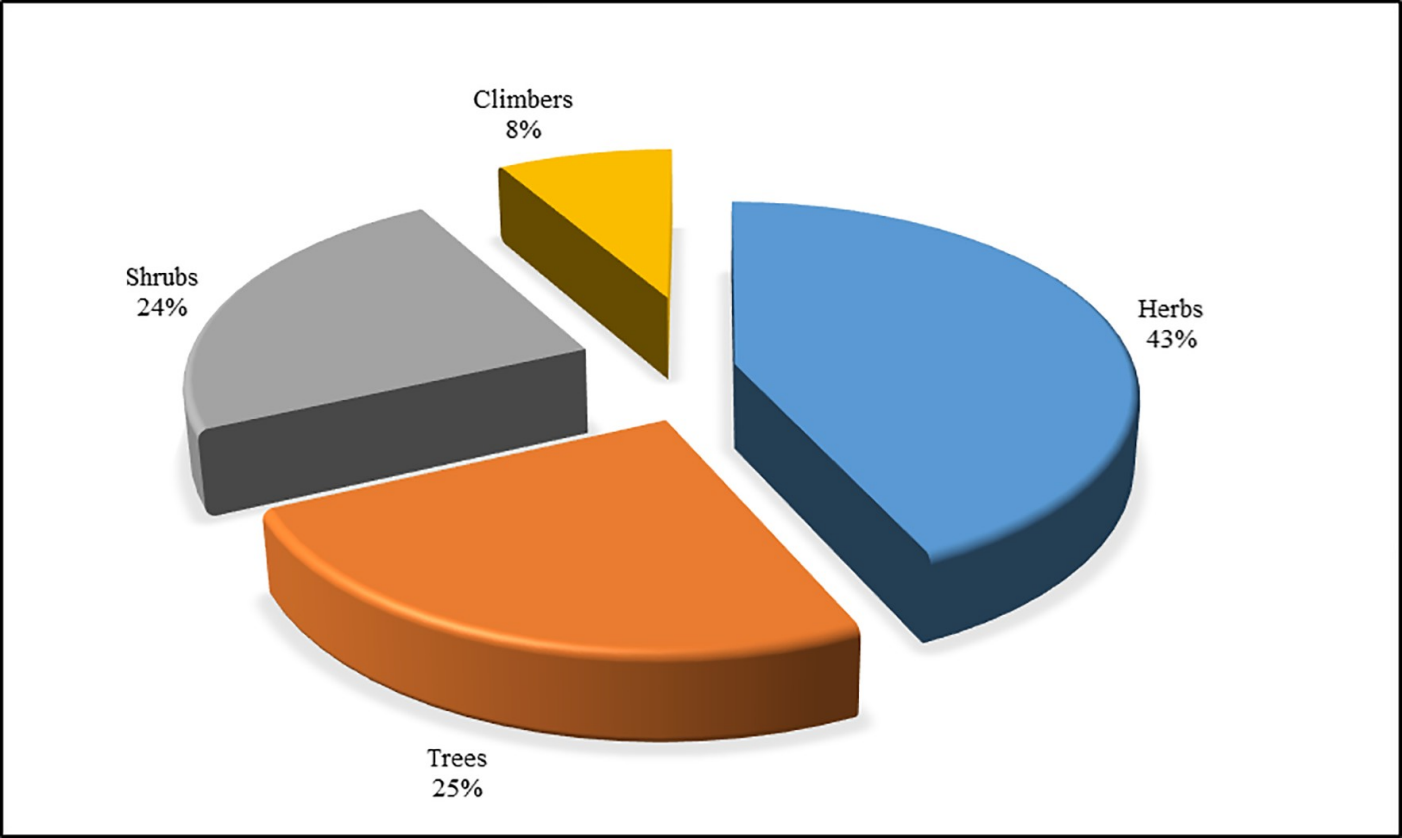
Distribution of wild edible plants in different life forms.

One species of fungi was also documented (four more folk names of edible fungi were recorded but excluded from the analysis since it was impossible to collect specimens and identify them). Herbs (43%) and trees (25%) were the dominant life forms, followed by shrubs (24%) and climbers (8%) (Fig 4). In terms of availability, about 65% of plant species were found in the nearby forest, grassland, wetland and agricultural land, while 30% were found in the higher mountain regions.

### Parts used and use categories of wild edible plants

**Fig 5:**
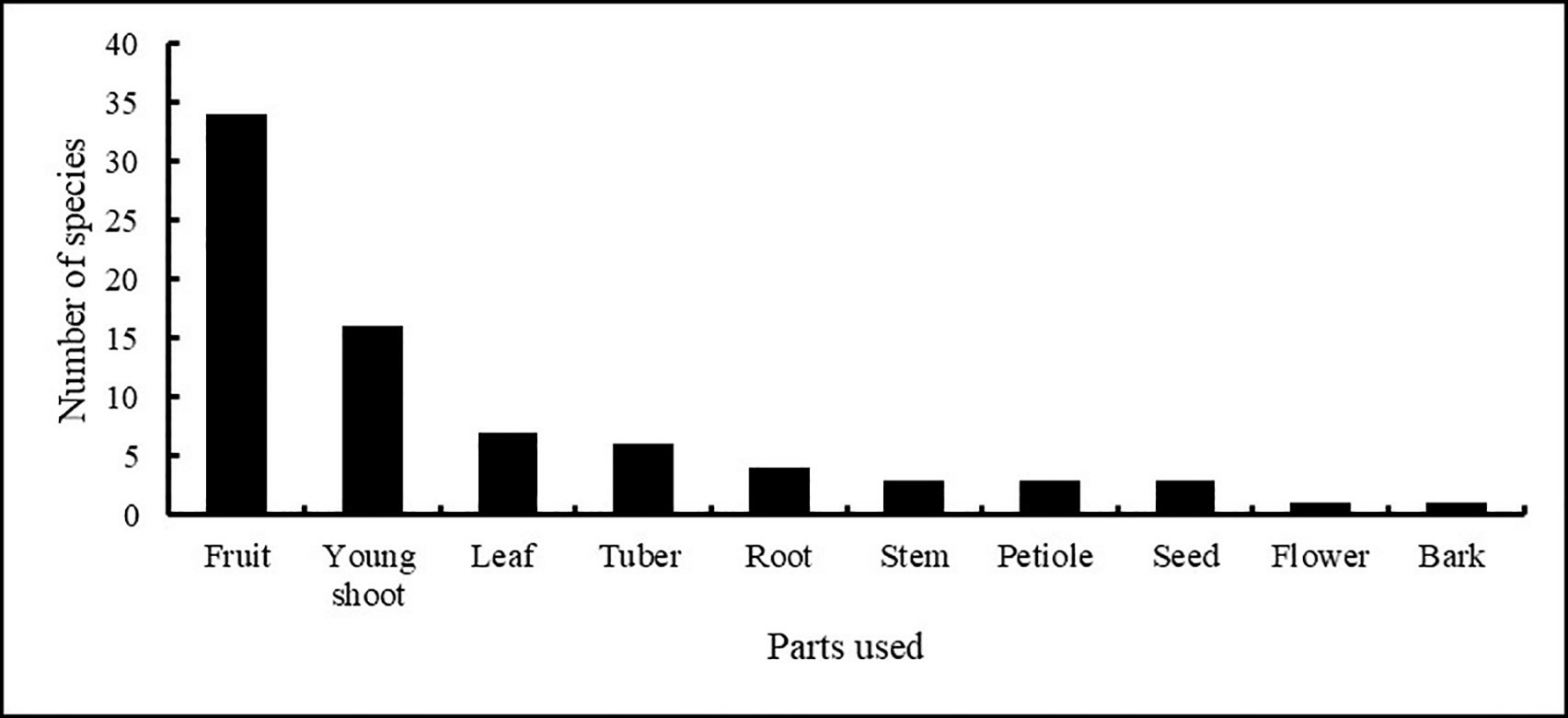
Use frequency of wild edible plant parts.

Almost all parts of WEPs were used for edible purposes. Fruits (34 species) were the most commonly used parts, followed by young shoots (16), leaves (7), tubers (6) and roots (4). Of all plant parts, flowers and bark were the least often used for edible purposes (Fig 5). Three categories of uses of WEPs were reported from the area, namely fruits, vegetables and seeds. Fruits were provided by 50% of species, while 41% species were used as vegetables. Seeds from *Cannabis sativa* and *Lindera neesiana* were also collected, used, and stored. Most of the fruit species were eaten raw once ripe, although some fruits were dried and stored for future consumption or used to make pickles. Green parts of plants were mainly consumed after cooking (*Arisaema costatum*, *Diplazium esculentum*, *Maianthemum purpureum* and *Phytolacca acinosa)* or in pickles (*Begonia dioca*, *Houttuynia cordata* and *Alsophila spinulosa*). Some species were also eaten raw as a snack (*Begonia dioca* and *Rheum australe*).

People used a range of different WEPs for various purposes other than food. The most common uses were as medicine (21 species); some species were used as fiber (4 species) and dye (1 species); other uses included the ornamental (2) and religious (5) (Table 1).

### RFC and preference ranking of wild edible plants

*Asparagus racemosus* (RFC = 0.97) was the most frequently consumed species, followed by *Lindera neesiana* (0.94), *Zanthoxylum armatum* (0.93), *Rubus ellipticus* (0.91) and *Berberis aristata* (0.89). Preference ranking of species with high RFC showed that *Asparagus racemosus* ranked top as the most preferred species with a total score of 46 out of 50 points. Likewise, *Rubus ellipticus* ranked first among the fruit species with a score of 41 (Table 2).

**Table 2:** Preference ranking among ten species with RFC value based on their use as perceived by the respondents.

S.N.	Plant species	Respondents										Total	Rank
		R1	R2	R3	R4	R5	R6	R7	R8	R9	R10		
**1**	*Asparagus racemosus* Willd.	5	5	5	4	3	4	5	5	5	5	46	**1st**
**2**	*Lindera neesiana* (Wall. ex Nees) Kruz	4	3	4	4	4	5	5	3	3	4	39	**3rd**
**3**	*Zanthoxylum armatum* DC.	3	3	5	3	4	3	2	1	2	1	27	7th
**4**	*Rubus ellipticus* Sm.	5	4	5	4	3	3	5	4	3	5	41	**2nd**
**5**	*Berberis aristata* DC.	3	5	1	2	4	5	1	5	4	2	32	**5th**
**6**	*Rumex nepalensis* Spreng.	4	2	1	2	1	3	2	3	1	2	21	10th
**7**	*Cinnamomum tamala* (Buch.-Ham.) Th. G. G. Nees	1	3	2	4	3	2	2	1	4	2	24	8th
**8**	*Viburnum mullaha* Buch.-Ham. ex D. Don	2	1	3	3	4	3	2	3	3	4	28	6th
**9**	*Himalayacalamus brevinodus *Stapleton	3	2	3	2	1	3	4	2	2	1	23	9th
**10**	*Tectaria coadunata* (Wall. ex Hook. & Grev.) C. Christensen	3	2	4	3	5	1	2	5	3	5	33	**4th**

### Traditional knowledge on edible plants in different informant groups

**Fig 6:**
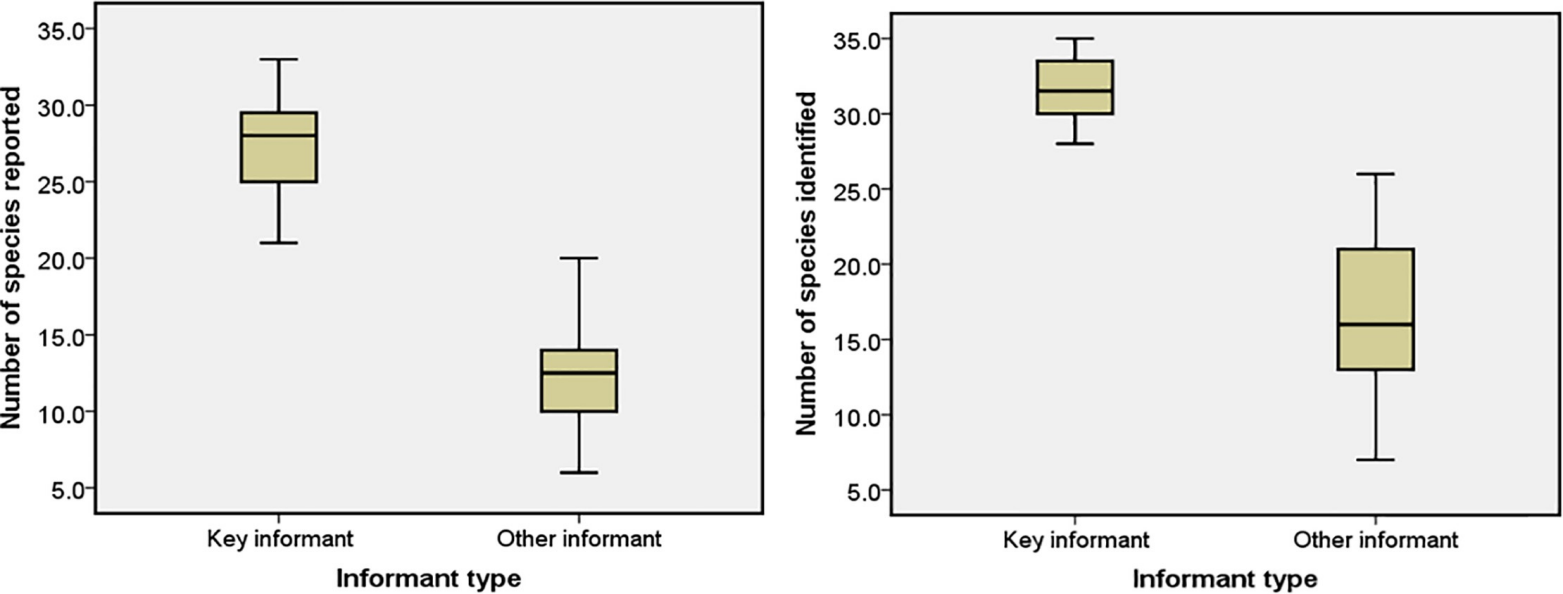
Median number of species reported and identified by key informants and others (median thick line inside box), 50 percentiles (box borders) as well as maximum and minimum values obtained.

The average number of plants listed by the informants was 14.4 (n = 70; mean). However, the key informants listed 27.4 plants on average (n = 8, mean), more than twice as many as the rest of the informants (12.4, n = 62, mean). The average number of plants identified was 18.7 (n = 70; mean), i.e. 30% of the plants presented to each informant. The difference between the groups with regard to the number of plants identified correctly was smaller than with regard to the number of plants listed, with key informants recognizing 32 on average (31.7, n = 8, mean) and the rest of the population recognizing 17 on average (17.0, n = 62, mean) (Fig 6).

There was no significant difference between genders in terms of the total number of species reported (men– 35.6 species, women– 35.5 species; Mann-Whitney U test; P > 0.05) or the total number of species identified (men– 37.5, women– 32.8; U = 520, Z = -0.952, P > 0.05).

**Fig 7:**
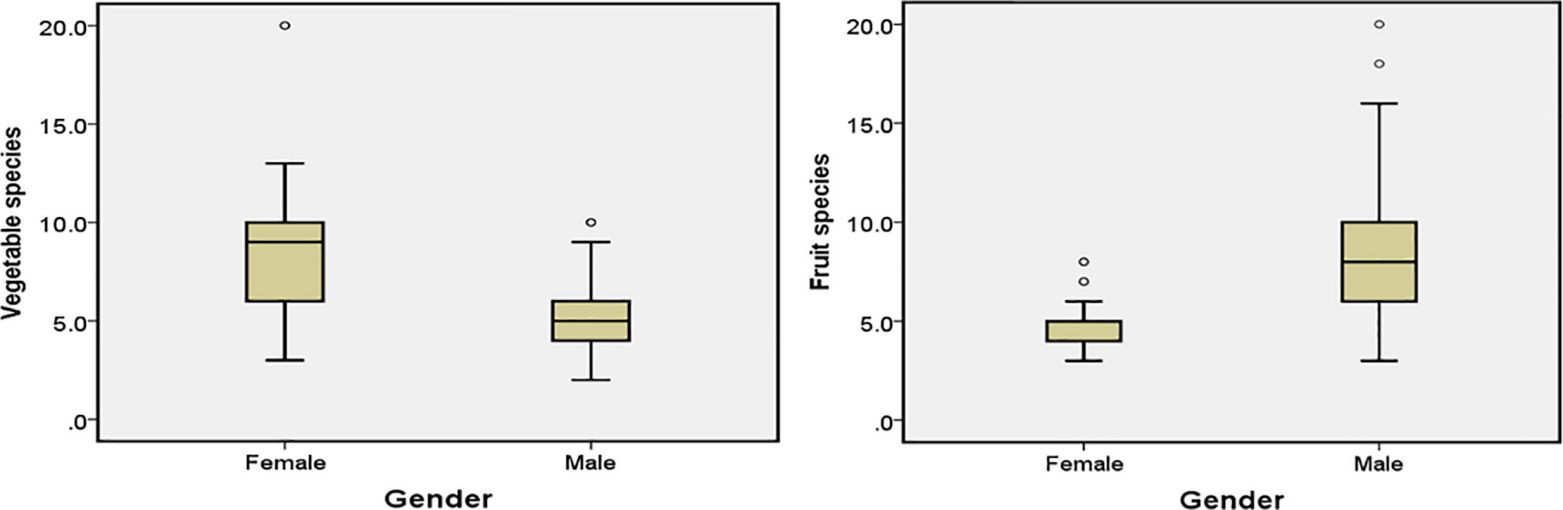
Differences between genders in reporting: Median number of plants reported (thick line inside box), 50 percentiles (box borders) as well as maximum and minimum values obtained.

**Fig 8:**
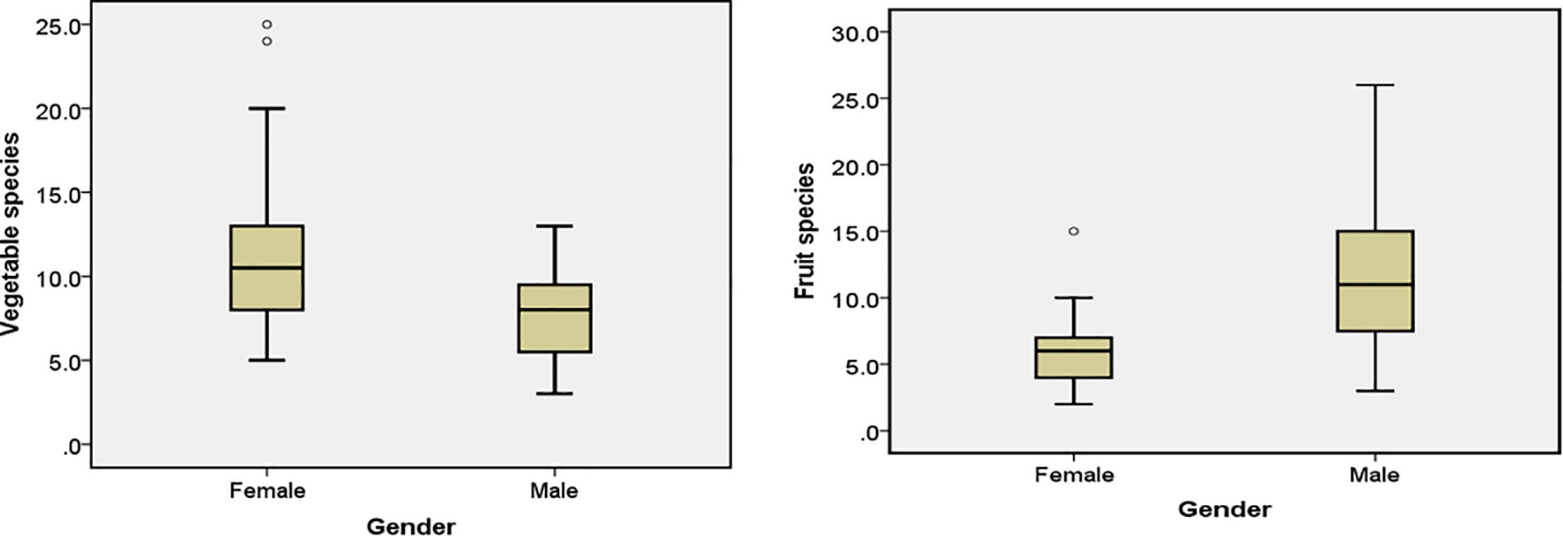
Differences between genders in identifying species: Median number of plants identified (thick line inside box), 50 percentiles (box borders) as well as maximum and minimum values obtained.

In terms of the number of species reported and identified according to category (vegetables and fruits), there was significant difference between genders (Mann-Whitney U test, U = 273, Z = -5.025, P < 0.05). Vegetable plant species were more often reported (mean: women– 9.0 species, men– 5.0 species; P < 0.05) and identified (women– 11.8, men– 6.2, P< 0.05) by women, whereas fruit plant species were more often reported (men– 9.35 species, women– 5.78 species; P < 0.05) and identified (men– 12.02 species, women– 6.81 species; P < 0.05) by men (Figs 7 and 8).

**Fig 9:**
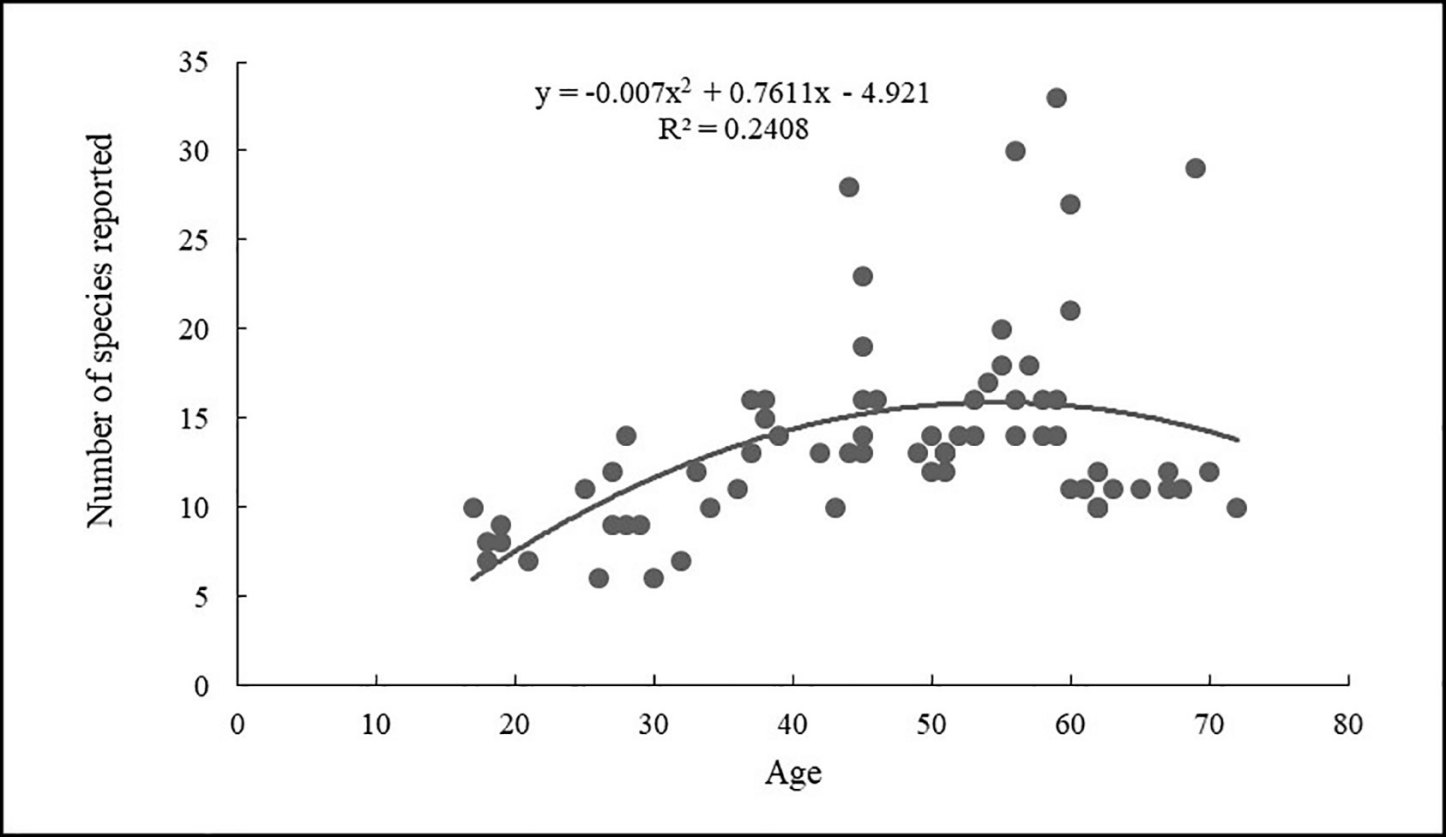
Informant age and number of species reported.

**Fig 10:**
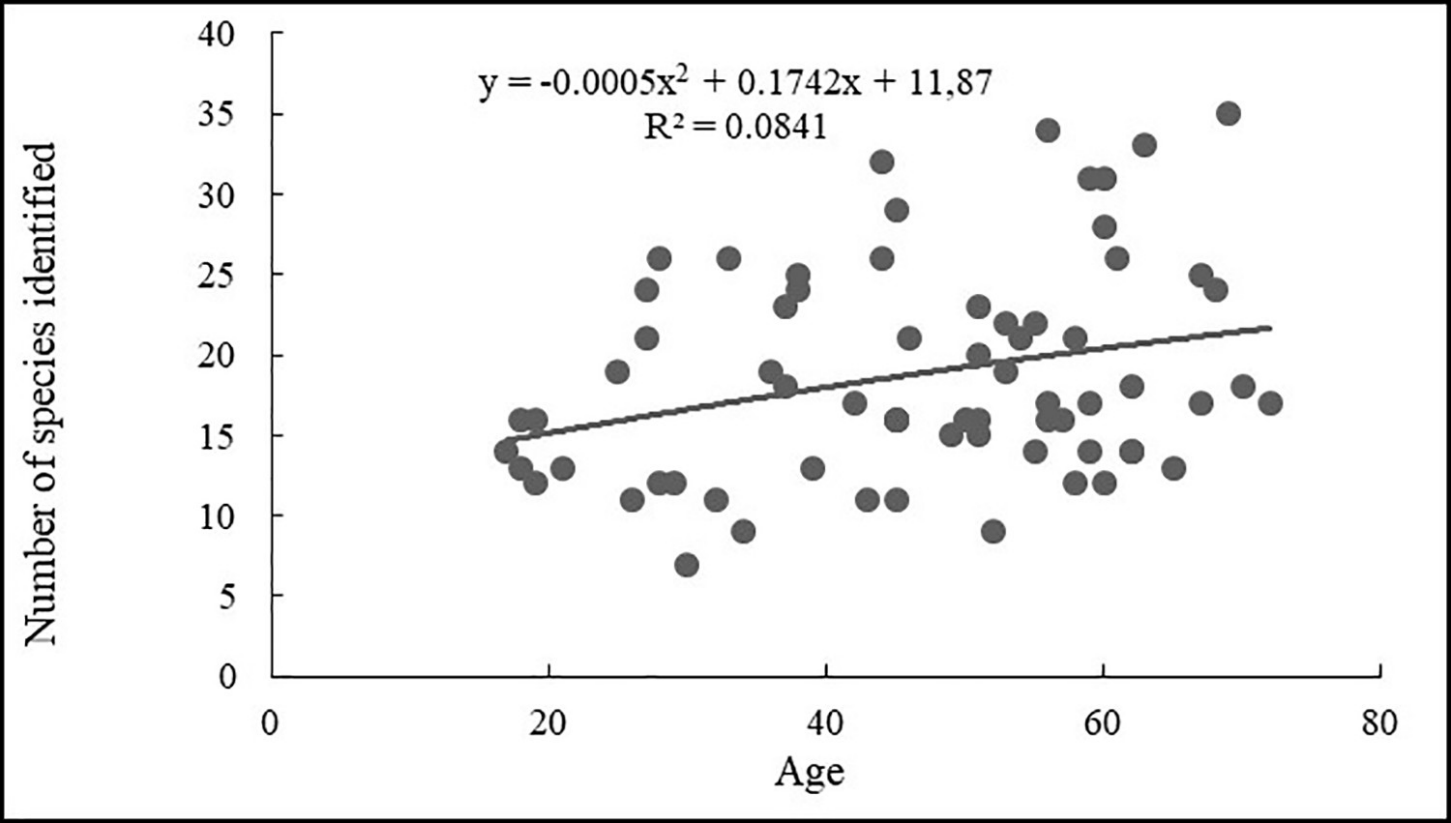
Informant age and number of species identified.

The relationship between age and the number of reported plant species is best explained by the polynomial curve (y = -0.007x^2^ + 0.7611x - 4.9218, R^2^ = 0.2408, p = 0.21; Fig 9), with maximum values for people aged 57, though the fit was still not significant. Similarly, the relationship between age and the number of identified plant species is also best explained (y = -0.0005x^2^ + 0.1742x + 11.87, R^2^ = 0.018, p = 0.14; Fig 10) with maximum values for people of above 50, though the fit was still not significant. Therefore, the age group above 45 (both male and female) reported and identified a greater number of plant species. Both vertical and horizontal transmission of traditional knowledge was reported in the present study, as respondents mentioned that they learned uses from grandparents and parents (59%) or from neighbors (28%). About 10% of the respondents also claimed to have learned about WEPs on their own while foraging and shepherding. These species include alpine species such as *Maianthemum purpureum*, *Koenigia polystachya* and *Berberis concinna*.

### Seasonality and harvesting techniques of WEPs

The species were collected and used mostly in monsoon and spring seasons. About 80% of the species were harvested in those seasons, whereas the winter season constituted only about 2% of total harvests. About 17% species were harvested in the autumn season (Table 1). It was observed that members of the community preserved and stored some plants in order to guarantee supply during off-peak seasons. The relative seasonal importance of WEPs shows that they were more important in the monsoon and pre-monsoon seasons than in the dry season. Most of the species were season-specific, that is, they were harvested and utilized in a particular period of the year. *Nephrolepis cordifolia* and *Urtica dioica* were found throughout the year. Some of the season-specific plant species collected and stored for later use were *Lindera neesiana* (spice, tea, medicine) *Brucea javanica* (pickle, medicine), and *Zathoxylum armatum* (spice, medicine and religious).

Plants were mainly harvested using three simple methods, namely digging tubers and roots (5%), plucking flowers, fruits and seeds (82%), and ground collection of fallen seeds and fruits (13%).

### Local perception on availability of WEPs in natural habitats

**Fig 11:**
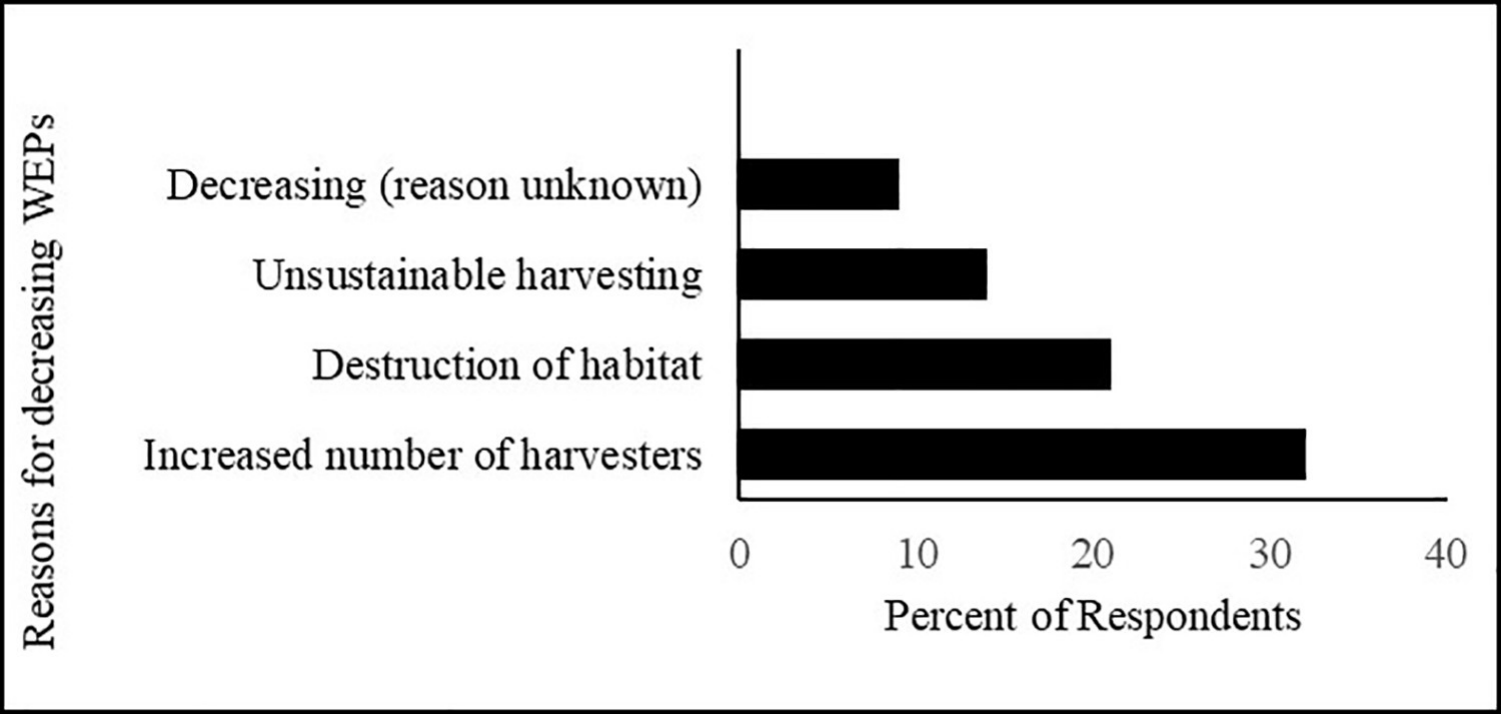
Local perception of the availability of WEPs.

The majority of the respondents (76%) reported that the availability of the WEPs was decreasing because of the increasing number of harvesters for particular species, unsustainable harvesting, and habitat destruction. The other reason behind decreasing availability was high market value due to medicinal properties (Fig 11), e.g., *Dactylorhiza hatagirea*, *Paris polyphylla* and *Rheum australe* Most edible fruits were harvested before they had reached maturity, and overharvesting was often reported for *Lindera neesiana* and *Zanthoxylum armatum*.

## Discussion

Globally, WEPs have been recognized as a key component in ecosystem-based adaptation and food scarcity copying strategy [3,52]. Similarly, WEPs always have a crucial role in diversifying cuisine as well as meeting food scarcity and nutritional needs in Nepal. In times of food shortage, people may resort to a larger number of species than normally [53]. This study focused on the traditional use of WEPs in rural areas of western Nepal.

### Diversity and use categories of WEPs

The species reported from this study possess a remarkably wide range of uses as compared to those reported by studies from, among others, Manang [30], Rupandehi [27] and some other parts of western Nepal (Kailali, Kanchanpur, Surkhet, Dang, Bardiya) [21,29]. This result was as expected, since the as *Gurung* ethnic group has wide knowledge on the use of natural resources [38,54] and the area is rich in biodiversity [39]. This difference in number of species between villages may be due to differences in altitude and variation in species composition–predominantly lower altitude species were reported in Khilang, whereas high altitude species dominated in Sikles. The reported dominancy of the botanical family Rosaseae is comparable with a study conducted in a similar location in the Manang district–a part of the Annapurna Conservation Area [30]. In terms of life forms, herbs were dominant, followed by trees, in contrast to Uprety et al. [22], who reported the opposite, and similarly to Aryal et al. [28]. Our results are in accord with Shrestha and Dhillion [16], where many of the food plants were herbaceous and produced fruits for consumption. There is also similarity in the use pattern of WEPs with Aryal et al. [28], as fruits are the most used plant parts, followed by vegetables.

The rich diversity of WEPs in the present study demonstrates that people in and around forest reserves possess information about local edible vegetation. This is because WEPs can provide both staple and complementary food for indigenous peoples and local communities and offer an alternative source of cash income for poor and countryside populations [14,28]. WEPs have the potential to greatly improve food security by providing alternative sources of affordable and nutritious food with the added advantage of being available all year round and the ability to grow in water-stressed areas and diverse environmental conditions [55]. It is apparent that people use the plants that are the most accessible or locally abundant, following the principles of optimal foraging theory [56]. It ought to be noted that availability is often conceptualized as the physical distance between from the home or community and the location where the plant grows in the wild, but it can also be considered in terms of seasonality, abundance, and price as well as access to markets, gardens, or natural areas where the plants are found [57].

According to Turreira-García et al. [18], the consumption of WEPs is premised on four reasons, namely i) hunger due to food scarcity, ii) spicing staple food, iii) nutria-medicinal value, and iv) preservation of cultural practices. In the present study, besides edible use, 21 species are also used for medicinal purposes. The nutria-medicinal value is a widespread factor among the local people. Species such as *Berberis aristata*, *Cirsium verutum*, *Dactylorhiza hatagirea*, *Lindera neesiana*, *Paris polyphylla* and *Zanthoxylum armatum* are the most used medicinal plants in the study area. The uses of most medicinal plants are similar to those shown in other studies conducted in Nepal [58]. Species such as *A*. *racemosus*, *B*. *javanica L*. *neesiana*, *Z*. *armatum* are regarded as multipurpose and mostly used for edible, medicinal and religious purposes.

Among vegetable plants, *A*. *racemosus* and *Rumex nepalensis* had high RFC scores, meaning that these species were highly valued. The availability of these two species close to settlements could have played a decisive role. Among the fruit species, *L*. *neesiana* had high RFC because of its multiple uses. *Z*. *armatum*, *B*. *aristata* and *Rubus ellipticus* are widely available species. Since knowledge on the use of WEPs differs from person to person, the output of the comparison showed that in many cases the informants perceived the plants differently, as it emerged from the scores they gave. The majority of the wild edible species in the area were eaten as extra food instead of being served as regular meals, as reported from Nepal [22,23,28] and elsewhere [59].

### Traditional knowledge in relation to informant, gender and age

Ecological as well as traditional knowledge is required for the identification, collection, and preparation of wild foods [60]. The distribution of such knowledge between individuals in a community is usually differentiated by gender, age or social role. Katul et al. [61] pointed out that in the mid-hills of Nepal knowledge of plant use follows a pattern determined by the available useful plants and sociocultural tradition of the particular area. The significant difference in the case of free listing as well as identification tests between key informants and other informants showed that key informants play a major role as ethnobotanical informants. These results are comparable with those obtained from Guatemala [18], where key informants recorded and identified a higher number of plant species than other informants. This may be due to their experience and association with WEPs. Key informants stated that living in a certain environment for a longer period of time increases the chances of using a resource and thus accumulating knowledge of local plants. Practical knowledge or skills in identifying plants are therefore greater in people who live or travel seasonally in resource-rich areas.

Although women are in general considered to be more knowledgeable about wild food plants than men [16], there was no significant difference between genders in relation to the total number of plants reported, meaning that both men and women were equally knowledgeable about WEPs.This finding is similar to Joshi et al. [23], who reported such a comparison from the Makawanpur district of central Nepal. Our finding also corroborates with Kang et al. [62], and it ought to be emphasized that both genders have a good knowledge of plants in traditional societies with deep knowledge. Bortolotto et al. [63] also pointed out in the Brazilian context that agricultural field activities and cattle handling are common to both genders, putting them in similar situations and providing them with an equal opportunity to know WEPs. The similar situation in the present study area played an important role in the acquisition of knowledge. Furthermore, the native vegetation near settlements gives both genders an equal opportunity to know WEPs.

While analyzing vegetable and fruit groups categorically, there was significant difference among the genders. Women were mostly responsible for collecting vegetable plants from nearby settlements, whereas men were involved in collecting plants from high altitude regions. As explained by Kujawska and Luczaj [64] in their study from Argentina, sometimes men could list more species, as they were not scared to go further into the forest.

As explained by the polynomial curve, informants above the age of 45 reported and identified more species of WEPs regardless of gender. Relatively older populations are the most knowledgeable groups, as reported from elsewhere [16,23,63]. However, sometimes younger people, if they are involved in foraging or shepherding, are knowledgeable particularly on wild fruit plants [22], meaning that the higher the exposure and association with the natural environment, the better the knowledge on the use of WEPs. Environmental changes, livelihood options, the availability and distribution of natural resources around residential areas, demographic characteristics, living period and availability of cultivated land are some of the factors that influence traditional knowledge on the use of natural resources, including WEPs [65–67].

### Seasonality and harvesting techniques of WEPs

Key informants explained that the time/season and frequency of harvesting vary from plant to plant depending on the availability of edible plants and their parts. The relatively high importance of WEPs in the rainy season coincides with the time when most species are re-sprouting, flowering, and fruiting, thereby increasing their availability. It ought to be noted that during the rainy season, most households are able to produce food from a range of crops and therefore have a wide range of choice, but wild plants are still important. Such seasonality of wild vegetable collecting, mainly in wetter periods, is widespread [66,68]. In the dry season, communities are solely dependent on stored food, and WEPs (especially vegetables) help to diversify the food intake. Species such as *Diplazium esculentum*, *Z*. *armatum* and *L*. *neesiana* are sundried and stored. On the other hand, young shoots of the *Dendrocalamus hamiltonii* are stored in the form of pickles. In the case of fruits, some are eaten when they are ripe and cannot be stored for long time, e.g. *Stauntonia angustifolia*, *Solena amplexicaulis* and *Pyracantha crenulata*. On the other hand, the fruits of species such as *Choerospondias axillaris* were pickled and stored for later use. The dried fruit powders of *Brucea javanica* and *Viburnum mullaha* are stored for a longer time and are some of the most popular WEPs. Although cultivated vegetable production is encouraging, local garden produce is often not sufficient to meet the demand for vegetables throughout the year. The collection and consumption of wild vegetables fills this gap [30].

All the methods used to harvest WEPs in this region can be termed as simple, and therefore they have less deleterious effects on the plant species. However, immature harvesting and overharvesting are still issues. Continued illegal collection (due to market value) has led to the depletion of many species such as *Paris polyphylla* and *Dactylorhiza hatagirea*. On the other hand, many plants may exhibit biological traits that potentially enable them to respond positively to sustained-yield harvest [69], and the most commonly collected species of wild foods generally tend to be common ubiquitous species in accord with the optimal foraging theory [56].

### Use, availability, and conservation of WEPs

Most of the available studies from various regions have found that socio-cultural factors are the main drivers of the reduced consumption of WEPs [70,71]. It has been strongly believed, mainly by indigenous peoples, that wild foods have a greater capacity to maintain the good health of those who depend on them [72]. Despite their accessibility and availability, the utilization of WEPs is challenged by numerous factors [55]. In the present study, most of the informants (76%) believed the availability of WEPs to be decreasing. The main reasons listed for decreasing availability are the increased number of harvesters for particular species and the destruction of natural habitats. FAO [73] identified the most widespread threats to WFPs use as overexploitation, habitat alteration, pollution, land-use change, and deforestation. Besides reduced availability, other reasons for not consuming WEPs could be limited knowledge about their nutrition and health benefits, the time involved in the collection and preparation of these foods, and the lower economic value of these resources.

As highlighted by Heywood [74], lack of information about the extent of use and importance (including economic) of WEPs in rural economies and the lack of reliable methods for measuring their contribution to farm households are some of the barriers to the promotion of WEPs on a larger scale, which is also true for the present study area.

However, a considerable number of respondents (23%) did not believe WEPs to be decreasing, citing valid reasons such as the migration of younger people to cities and only older people being left in the villages, unable to collect plants from the highlands. Another valid reason they cited was the increase in forest cover preventing a decrease in WEPs.

Since threats to biodiversity in general are also threats to WEPs, both in Nepal and elsewhere, overharvesting and habitat destruction should be controlled for better conservation of WEPs using various means such as increasing awareness and monitoring. There is no controlled access for collecting WEPs for household use, even though the study area is part of a conservation area. Nevertheless, illegal harvesting of traded medicinal plants and timber is reported as the permits are required for harvesting these items. The ACA has a different model of conservation and management of natural resources. The Conservation Area Management Committees (CAMCs) are responsible for monitoring illegal activities, and these community conservation groups should also be held more responsible for conservation of WEPs. The CAMC can decide on the sustainable use of resources on need basis thus supporting local livelihood and culture. The present management arrangements favor the sustainability of WEP. The Annapurna Conservation Area Project (ACAP) supports the work of CAMC, thereby establishing a strong partnership with local communities.

The species with potential for cultivation in farmlands can be identified based on available cultivation techniques, market demands, and traditional knowledge of their preferences. It is generally accepted that the lack of suitable data for prioritizing conservation action greatly hampers plant conservation efforts [75]. Since almost all of the wild edible plant species in this area have uncertain conservation status (not evaluated), this should be the priority of conservation efforts.

### Archaic features of WEPs use

The use of WEPs in the study area has some archaic features. One of them is drying wild vegetables for winter, a tradition now preserved only in a few countries in the world [76]. Another feature is the use of a relatively large number of underground parts of plants (10 species, i.e. 14%), which is a feature of hunter-gatherer societies and has disappeared in most agricultural food systems [9]. For comparison, in similar studies from SW and S Asia usually 2–7% species are eaten for underground parts (e.g. [22,28,66,77,78]). Yet another is the use of a large number of species of ferns. Ferns are typical woodland plants, and their use for consumption may be seen as a vestige of ancient hunter-gather practices, as pointed out by Pieroni et al. [79]. Although their use is quite widespread in mountainous parts of south and eastern Asia [10], the taxa used in different parts of the continent have not been fully identified.

## Conclusion

This study revealed that traditional knowledge regarding use, distribution and collection of WEPs is still well-maintained. The preservation of this knowledge appears to be the result of the continued reliance of local communities on WEPs resources. The results also revealed that many wild species are under growing pressure from various anthropogenic factors. Thus public awareness and community-based management need to be encouraged.The findings suggest the need for further investigation into nutritional profiles and processing methods of all the species. Efforts to conserve biodiversity and preserve traditional food systems need to be combined and enhanced for the benefit of posterity. Further studies providing this data would greatly assist in promoting the involvement of local people in managing their resources. Our study also helped enrich the herbarium, offering permanent herbarium records and specimens for determination and quick botanical reference in the future.

## Supporting information

S1 File Questionnaires used for collection of information pertaining to wild edible species (translated from Nepali language). (DOCX)
