# Supplementary material for: Foods from the wild: Local knowledge, use pattern and distribution in Western Nepal
Source: PLoS One. 2021 Oct 21;16(10):e0258905. doi: 10.1371/journal.pone.0258905 (PMC8530312; doi:10.1371/journal.pone.0258905)
Supplement: S1 File — (DOCX) [file pone.0258905.s001.docx]

**Questionnaires used for collection of information pertaining to wild edible species**

**(Translated from Nepali Language)**

Date: …………………

Locality: …………

Name of the respondent: ………………………

Age: …………..

Gender: a) Male b) Female

Occupations: ………

Education level: ………………………………

1. What are the plants used for edible purpose that are collected from the wild?

Local name of the species: ……………………

Identifying characteristics of the species: ……….…………

Parts used: ……………..……………….

Used for: ………………………………………..……..………………

Method of use: ………………………….………………….…………..

Time of harvesting: …………………………………..………………

Area of collection: ……………………………………….………………

2. What is the availability (past and present trends) of the species in the wild?

3. Which groups of the community commonly collect and use wild edible species (gender and age wise)?

5. What are the methods and seasons for harvesting those wild edible species?

6. How did you know the particular use of this edible species?

7. Does the species have additional use other than food? If yes, what is that?
